# Sodium–glucose cotransporter 2 inhibitors ameliorate glutathione cysteine ligase modifier-mediated oxidative stress and subsequent ferroptosis in proximal tubules of diabetic kidney disease

**DOI:** 10.1080/13510002.2025.2528334

**Published:** 2025-07-28

**Authors:** Yi-Chun Tsai, Jiun-Chi Huang, Ping-Shaou Yu, Mei-Chuan Kuo, Ling-Yu Wu, Wei-An Chang, Shang-Jyh Hwang, Ya-Ling Hsu

**Affiliations:** a School of Medicine, College of Medicine, Kaohsiung Medical University, Kaohsiung, Taiwan; b Division of General Medicine, 10.13039/501100011645 Kaohsiung Medical University Chung-Ho Memorial Hospital, Kaohsiung Medical University, Kaohsiung, Taiwan; c Division of Nephrology, Kaohsiung Medical University Hospital, Kaohsiung Medical University, Kaohsiung, Taiwan; d Drug Development and Value Creation Research Center, Kaohsiung Medical University, Kaohsiung, Taiwan; e Department of Internal Medicine, Kaohsiung Municipal CiJin Hospital, Kaohsiung, Taiwan; f Department of Internal Medicine, Kaohsiung Municipal Siaogang Hospital, Kaohsiung Medical University, Kaohsiung, Taiwan; g Graduate Institute of Clinical Medicine, College of Medicine, Kaohsiung Medical University, Kaohsiung, Taiwan; h Division of Pulmonary and Critical Care Medicine, Kaohsiung Medical University Hospital, Kaohsiung Medical University, Kaohsiung, Taiwan; i Graduate Institute of Medicine, College of Medicine, Kaohsiung Medical University, Kaohsiung, Taiwan

**Keywords:** Sodium–glucose cotransporter 2 inhibitor, proximal tubule, oxidative stress, ferroptosis, glutathione cysteine ligase modifier, diabetic kidney disease, transcriptome analysis, N-acetylcysteine, ferrostatin-1

## Abstract

**Objectives:**

Diabetic kidney disease (DKD) is a major cause of end-stage kidney disease. The precise molecular mechanism of ferroptosis, an iron-dependent and non-apoptotic form of regulated cell death, remains poorly understood in DKD, as does the impact of sodium-glucose cotransporter 2 inhibitors (SGLT2i) on ferroptosis-mediated DKD.

**Methods:**

This study used bulk RNA sequencing, in vitro and in vivo models, and human kidney samples to explore the molecular mechanisms involved in oxidative stress and ferroptosis in the proximal tubule (PT) of DKD.

**Results:**

High glucose (HG) induced features of ferroptosis in HK-2 cells. Transcriptome analysis of primary PT cells from diabetic patients indicated that glutathione cysteine ligase modifier (GCLM) subunit is involved in ferroptosis. Immunohistochemical staining revealed that db/db mice and diabetic patients had lower glutathione peroxidase 4 and GCLM expression in the PT. Suppression of GCLM enhanced ferroptosis, whereas GCLM overexpression mitigated HG-induced ferroptosis in HK-2 cells. Antioxidants reduced oxidative stress and ferroptosis in both in vitro and in vivo models of DKD. Furthermore, SGLT2i attenuated PT ferroptosis in these models and improved DKD by increasing GCLM expression.

**Conclusion:**

SGLT2i reduced ferroptosis in PT by boosting GCLM expression, thereby slowing DKD progression, revealing that GCLM has the potential against DKD.

## Introduction

Diabetes poses a growing threat to human health and is a significant public health concern [1]. Diabetic kidney disease (DKD) is the leading cause of end-stage kidney disease (ESKD) globally, accounting for 40∼50% of all ESKD patients [1]. Consequently, preventing the onset or progression of DKD may reduce the incidence of ESKD. Multiple pathophysiological factors contribute to the development and progression of DKD [2], with hyperglycemia and hypertension being key contributors [3]. Controlling hyperglycemia is essential for preventing and slowing diabetes-related complications, but it alone cannot fully stop the disease progressing. Other factors like lifestyle habits and dyslipidemia also significantly contribute [4,5]. Therefore, additional therapeutic drugs targeting DKD are urgently required.

Intrarenal oxidative stress is crucial for the initiation and development of DKD [6]. Ferroptosis is a form of regulated cell death that is dependent on reactive oxygen species (ROS) and is characterized by the accumulation of iron and lipid hydroperoxides, as well as subsequent reactive aldehydes such as malondialdehyde (MDA) [7]. Free iron acts as a cofactor for lipid hydroperoxide enzymes and ROS production [8]. Additionally, iron directly produces ROS via the Fenton reaction, thereby triggering oxidative damage [8]. The antioxidant glutathione peroxidase 4 (GPX4) protects cells from oxidative stress and phospholipid hydroperoxide-mediated ferroptosis [7]. The morphology of ferroptotic cells is primarily characterized by changes in the mitochondria, including condensation or swelling, increased membrane density, and reduced mitochondrial cristae [7,9]. Recently, ferroptosis has been implicated in various kidney diseases, including acute kidney injury, chronic kidney disease, polycystic kidney disease, and renal cell carcinoma [9]. Although little evidence has revealed the impact of ferroptosis on DKD [10,11], whether ferroptosis contributes to DKD by inducing kidney tubular injury remains unclear.

Sodium–glucose cotransporter 2 inhibitors (SGLT2i) are a class of anti-hyperglycemic medications approved for the treatment of type 2 diabetes (T2D) [12,13]. They function by blocking the renal reabsorption of glucose through the inhibition of SGLT2, the main sodium-coupled glucose transporter in the proximal tubules (PTs), resulting in glycosuria and reduced blood glucose levels [12]. Beyond its capacity to enhance urinary glucose excretion and manage glycemia, SGLT2i also possesses additional properties that could be beneficial for renoprotection in DKD [13,14] and has been demonstrated to reduce the risk of composite kidney outcomes including macroalbuminuria, doubling of serum creatinine, initial dialysis, or kidney death in T2D patients [15–17]. The CREDENCE trial also showed consistent benefits of canagliflozin, an SGTL2i, for the treatment of DKD in 2019 [18]. Moreover, the DAPA-CKD trial included patients with chronic kidney disease (CKD), both with and without T2D, and found that treatment with SGTL2i dapagliflozin reduced the composite kidney end point by 31% [19]. SGLT2i variants also exhibit antioxidative properties [20]. Proximal tubular epithelial cells (PTECs) are the primary targets of injury in glucose-induced metabolic disorders, and changes in PT occur early in diabetic kidneys [5,21]. Thus, the aim of this study was to investigate the pathophysiological role of ferroptosis in the PT of DKD and whether SGLT2i could rescue DKD by suppressing ROS and consequent ferroptosis.

## Materials and methods

### Cell culture

PTECs of a T2D patient (Lonza CC-2925) and a healthy individual (Lonza CC-2553) were purchased from Lonza (Lonza Walkersville Inc., MD, USA) and cultured using Clonetics REGM BulletKit (Lonza CC-3190). Human kidney-2 (HK-2) cells (ATCC CRL-2190) were cultured in keratinocyte serum-free medium with 2% fetal bovine serum (FBS, Cat No. 26140079, Gibco, ThermoFisher Scientific, MA, USA). The cells were maintained in either normal glucose (NG, 5.5 mM, Cat No. G7021-1 kg, Sigma-Aldrich, MA, USA) or high glucose HG (25 mM, Cat No. G7021-1 kg, Sigma-Aldrich, MA, USA), with or without Ferrostatin-1 (Fer-1, 2 μM, Cat No. SML0583, Sigma-Aldrich, MA, USA), catalase (0.4 μM, Cat No. C1345, Sigma-Aldrich, MA, USA), or dapagliflozin (DAPA, 2 μM, Cat No. SC-364481B, Santa Cruz, TX, USA) for the specified durations. Different doses of Fer-1 and DAPA were tested to evaluate toxicity of HK-2 cells by WST-1 assay, and found no obvious toxicity in HK-2 cell viability across 1 μM, 2 μM to 5 μM (Figure S1(A,B)). According to the results of WST-1 assay and previous studies [22,23], Fer-1 (2 μM) and DAPA (2 μM) were used to treat HK-2 cells. There were at least three independent experiments in each cell group.

### RNA sequencing and bioinformatics analysis

Bulk RNA sequencing (RNA-seq) of harvested cells was conducted by Welgene Biotechnology Company (Welgene, Taipei, Taiwan) and analyzed using bioinformatics, as described in our previous study [23]. Briefly, primary PTECs from one T2D patient and one healthy individual (Lonza Walkersville Inc., USA) were cultured in Clonetics REGM BulletKit. RNA samples from primary PTECs from one T2D patient and one healthy individual were collected and profiled by next-generation sequencing [24]. Differentially expressed mRNAs in primary PTECs between a T2D patient and a healthy individual were identified with a threshold of >1.5-fold change and >0.3 fragments per kilobase of transcript per million (GSE185586). In addition, we used two public GEO databases to support our bulk RNA-seq data [25,26]. Sas et al. [25] collected RNA samples from the glomerular-deprived kidney cortex of five db/db and five db/m mice (GSE86300) to validate ferroptosis in DKD. Uehara-Watanabe et al. collected RNA samples from isolated tubular epithelia from three db/db mice, three db/m mice, and three db/db mice taking SGLT2i (GSE185801) to validate the role of SGLT2i in ferroptosis in DKD [26]. The functions and interactional networks of the altered mRNAs were evaluated using the Database for Annotation, Visualization, and Integrated Discovery (DAVID) or STRING database (https://string-db.org/).

### Quantitative real-time PCR analyses

The expression of GPX4 mRNA was assessed using SYBR Green on a StepOnePlus quantitative real-time PCR (qRT-PCR) system (Applied Biosystems, Foster City, CA, USA), as previously described [24]. The expression of glutathione cysteine ligase modifier subunit (GCLM) mRNA was examined using the TaqMan Fast Advanced Master Mix following the manufacturer’s protocol. (Applied Biosystems, Thermo Fisher Scientific, MA, USA). The relative expression of a specific mRNA was normalized to that of the internal control GAPDH. The primer sequences used for real-time PCR were as follows:

TaqMan Fast Advanced Master Mix:

GCLM: CGCACAGCGAGGAGCTTCATGATTG (Cas Hs00157694_m1);

GAPDH: CGCTGCCAAGGCTGTGGGCAAGGTC (Cas.Hs02786624_g1)

SYBR Green on the StepOnePlus qRT-PCR system:

GPX4 forward, 5′-CCGTGTAACCAGTTCGGGAA-3’; reverse, 5′-GCCCTTGGGTTGGATCTTCA-3′; GAPDH forward, 5′-GAGTCAACGGATTTGGTCGT-3′; reverse, 5′-TTGATTTTGGAGGGATCTCG-3′.

### Transient transfection

GCLM siRNA (20 nM, Cat No. L-011670-01-0005, Dharmacon, CO, USA), GCLM cDNA plasmid (4 ng, EX-M0714-M92, Genecopoeia, MD, USA), normal control (NC) siRNA (20 nM, Cat No. D-001810-10-05-20, Dharmacon, CO, USA), and NC cDNA plasmid (4 ng, Cat No. EX-NEG-M92, Genecopoeia, MD, USA) were transfected into cells using Lipofectamine RNAMAX or Lipofectamine 2000 transfection reagent (ThermoFisher Scientific) following the manufacturer’s protocols. The efficacy of GCLM siRNA knockdown and overexpression was confirmed by qRT-PCR and validated by western blot analysis. We used the OmicsLink expression clone data sheet EX-M0714-M92 for GCLM overexpression and EX-NEG-M92 as a negative control (GeneCopoeia, MD, USA). The sequences of the GCLM siRNA, NC siRNA, GCLM cDNA, and NC cDNA are listed in Table S1.

### Western blot analysis

The total protein of conditioned HK-2 cells was extracted using RIPA (radioimmunoprecipitation assay) lysis buffer (EMD Millipore, USA). The denatured proteins were separated by 9-11% SDS-PAGE electrophoresis, and then transferred onto a polyvinylidene difluoride membrane. After blocking, the membranes were immunoblotted with specific primary antibodies followed by secondary antibodies. Antibodies against GCLM (Cat No. 14241-1-AP, Proteintech, Chicago, IL, USA) and GAPDH (Cat No. MAB374; Millipore, Billerica, MA, USA) were utilized. The blot signals were captured using a Proteinsimple + Fluorchem Q system (Alpha Innotech, USA), and densitometry analysis was performed using ImageJ software (http://imagej.org/).

### Iron assay

The intracellular total iron levels were assessed using an iron assay kit (Cat No. SI-MAK025-1KT, Sigma-Aldrich, MA, USA). Cultured cells (2 × 10^6^ cells) were harvested and homogenized in iron assay buffer. The samples were then centrifuged at 16,000 × *g* for 10 min, and 100 μl of the supernatant from each cell lysate was combined with 5 μl of iron assay buffer and 5 μl of iron reducer. Then, 100 μl of the iron probe was added to each well containing both standard and test samples, and the reaction was incubated for 60 min at 25°C. Total iron levels were quantified using an enzyme-linked immunosorbent assay (ELISA) reader at a wavelength of 593 nm.

### Measurement of cellular MDA

MDA, the product of lipid peroxidation, was measured using a lipid peroxidation assay kit (Cat No. MAK085-1KT, Sigma-Aldrich, MA, USA). Cultured cells (2 × 10^6^ cells) were harvested and homogenized on ice in 300 µL of MDA Lysis Buffer containing 3 µL of butylated hydroxytoluene and then centrifuged at 13,000 × *g* for 10 min. MDA levels in the supernatant were assessed using thiobarbituric acid reaction at a wavelength of 532 nm. The MDA concentration of each sample was calculated based on a standard curve.

### Measurement of mitochondrial membrane potential

Mitochondrial membrane potential (MMP; ΔΨm) was measured using rhodamine 123 staining (Cat No. R8004, Sigma-Aldrich, St. Louis, MA). Briefly, live cells (7 × 10^3^) were incubated for 30 min in a medium containing the desired fluorescent substrate (10 µg/ml rhodamine 123) for 10 min at 37°C in an incubator containing 5% CO_2_. The fluorescence intensity of rhodamine 123 was measured using a fluorescence reader with excitation and emission wavelengths of 485 and 528 nm, respectively.

### Detection of glutathione and ROS

Glutathione levels were measured using a cellular glutathione detection assay kit (Cat No. 13859; Cell Signaling Technology, MA, USA) following the manufacturer's instructions. Fluorescence intensity was recorded using a fluorescence reader at excitation and emission wavelengths of 380 and 485 nm, respectively.

To assess the ROS levels, the ROS-Glo H_2_O_2_ Assay system (Cat No. G8820, Promega, WI, USA) was used according to the manufacturer’s instructions. Cultured cells were trypsinized, counted, and seeded in a 96-well plate at a density of 7 × 10^3^ cells/well in 80 μL of medium. Next, 20 μL of H_2_O_2_ substrate was added to each well and the plate was returned to the cell culture incubator for 45 min. ROS-Glo detection solution (100 μL) was added to each well and incubated for 20 min at room temperature on a rocker. Luminescence was measured using a fluorescence microplate reader (Biotek FLx800; Vermont, USA).

### Cell viability assay

Cell viability was assessed using a WST-1 Cell Proliferation Assay (ClontechTM Laboratories Inc., Mountain View, CA, USA). HK-2 cells (7 × 10^3^ cells/well) were seeded in 96-well plates and treated with dapagliflozin (2 μM) for 48 h. The cells were incubated with the WST-1 substrate at 37°C for 1 h, and absorbance was measured at 450 nm using an ELISA reader.

### Cell death detection

Cells were cultured in 96-well plates and stained with Hoechst 33342 (6 μM, Cat No. 62249, Thermo Scientific, USA) and propidium iodide (PI) solution (40 μg/ml, Cat No. SI-P4864; Sigma-Aldrich, USA) at 37 °C for 30 min in the dark. The staining results were observed using a fluorescence microscope (Nikon eclipse te2000-s, Japan), and images were collected. Double staining (Hoechst 33342/PI double-stained cells) was used to count ferroptotic cells.

### Animal experiments

All animal protocols were approved by the Committee for the Care and Use of Laboratory Animals at Kaohsiung Medical University, Kaohsiung, Taiwan (approval no. 110100). The in vivo sample size was determined based on previous studies and statistical power calculations (G Power software 3.1.9.7) to ensure adequate detection of the expected effects. We aimed for a sample size sufficient to achieve a power of at least 95% with an alpha level of 0.05, and the result of G power revealed that the sample number for adequate detection of expected effects was two at least. Pathogen-free 6-week-old male db/m mice (normal control, weight, 18–22 g, *n* = 16) and db/db mice (DKD, weight, 29–32 g, *n* = 20) were purchased from the National Laboratory Animal Center in Taiwan and were housed in a specific pathogen-free environment with controlled temperature (22–24˚C) and humidity (45-55%). The mice were maintained on a 12–h light/dark cycle and had ad libitum access to autoclaved water. Our animal center used gamma rays to maintain sterilized rodent chow, and proteins and vitamins were not denatured throughout the experiment. Oral gavage was used for treatment. At 8 weeks of age, all mice were randomly assigned into eight groups: (1) db/m mice treated with normal control (NC, water 6 ml/kg/day), (2) db/m mice treated with antioxidant (*N*-acetylcysteine [NAC], 200 mg/kg/day; [600 mg NAC dissolved in 6 ml water] for 6 weeks, Novatis, Swiss), (3) db/db mice treated with NC (water 6 ml/kg/day), (4) db/db mice treated with NAC (200 mg/kg/day for 6 weeks), (5) db/m mice treated with NC (water 1 ml/kg/day), (6) db/m mice treated with dapagliflozin (DAPA, 1 mg/kg/day; DAPA 1 mg freshly prepared by suspending in 10 μl ethanol and adding water to 1 ml prior to use, Cat No. SC-364481B, Santa Cruz, TX, USA) for 6 weeks, (7) db/db mice treated with NC (water 1 ml/kg/day), and (8) db/db mice treated with DAPA (1 mg/kg/day for 6 weeks). There were five mice in each group, except for db/m mice treated with NAC and DAPA (negative control group, *n* = 3). The doses of NAC and DAPA treated in the mice were based on previous studies [27–29]. The overall condition of the mice, including vocalization, respiratory difficulty, weight loss, abnormal behavior, and physical appearance, was monitored daily. In addition, we did not observe any dehydration or hypotension symptoms, such as dry skin and poor spirit, in the mice after DAPA administration. Body weight and blood glucose levels were monitored and 24-h urine samples were collected weekly. The humane endpoint of the mice was defined as a body weight loss of >20%, with none of the mice reaching this endpoint during the experiment, and mice remained active throughout all observations. At the endpoint of the experiment, all mice were db/m mice treated with water (weight, 20–22 g), db/m mice treated with NAC (weight, 22–26 g), db/m mice treated with DAPA (weight, 24–25 g), db/db mice treated with water (weight, 39–44 g), db/db mice treated with NAC (weight, 39–43 g), and db/db mice treated with DAPA (weight, 33–37 g). All mice were euthanized with 40% carbon dioxide at 14th weeks. Death was confirmed by respiratory and cardiac arrest along with pupil dilation, persisted for over 10 min. Following confirmed mortality, blood was collected via retro-orbital bleeding (1 ml) and the kidneys were harvested and fixed in 4% paraformaldehyde (Cat No. 3933, J.T. Baker, USA). for formalin-fixed and paraffin-embedded blocks. All samples were stored at −80°C for further analysis.

### Measurement of albuminuria and urinary neutrophil gelatinase-associated lipocalin and kidney injury molecule 1 in the mice

Urinary albumin levels were assessed using the immunoturbidimetric assay with Tina-quant Albumin Gen.2 (ALBT2, Roche, USA), and the levels of kidney injury molecule 1 (KIM-1; Cat No. MKM100, R&D Systems, MN, USA) and neutrophil gelatinase-associated lipocalin (NGAL; Cat No. MLCN20, R&D Systems, MN, USA) in the urine of mice was measured using ELISA kits. All urinary parameters were corrected for urinary creatinine (Cr). The urinary Cr concentrations were determined using an enzymatic method (Roche Diagnostics, Mannheim, Germany).

### Human study participants

Kidney sections were obtained from five patients with DKD scheduled for biopsies at Kaohsiung Medical University Hospital (KMUH) from November 2016 to August 2020 and five patients with upper tract urothelial carcinoma (UTUC) who underwent nephrectomy at Kaohsiung Municipal Ta-Tung Hospital from October 2018 to August 2023. The information of demographic characteristics including age, sex, and duration of diabetes was obtained from interviews and medical records of subjects at enrollment. Blood and single-point urine samples were collected for biochemical analysis. Urinary protein levels were assessed using turbidimetric method (Roche Diagnostics, Mannheim, Germany). The serum urea nitrogen and urinary Cr concentrations were determined using an enzymatic method (Roche Diagnostics, Mannheim, Germany). Serum Cr levels were measured using the compensated Jaffé method with a Roche/Integra 400 analyzer (Roche Diagnostics, Mannheim, Germany). Estimated glomerular filtration rate (eGFR) was calculated using the Chronic Kidney Disease Epidemiology Collaboration (CKD-EPI 2021) equation [30]. This study was approved by the Institutional Review Board of Kaohsiung Medical University Hospital (KMUHIRB-G(I)−20160017, KMUHIRB-G(I)−20150044, and KMUHIRB-20130089). All the participants provided written informed consent in accordance with the Declaration of Helsinki.

### Periodic acid-Schiff stain, terminal deoxynucleotidyl transferase dUTP nick-end labeling assay and immunohistochemistry stain

The features of the mouse kidneys were detected using periodic acid-Schiff (PAS) staining, according to the manufacturer’s protocols (Cat No. PAS-IFU, ScyTEK, UT, USA). Cell death in mouse kidneys was evaluated using the terminal deoxynucleotidyl transferase dUTP nick-end labeling (TUNEL) assay (Cat No. 11684817910, Roche, Germany). The levels of GPX4 and GCLM in the mouse and human kidney sections were detected using anti-GPX4 (1:100, Cat No. ab125066, Abcam, Cambridge, UK), and GCLM (1:100, Cat No. 14241-1-Ap, Proteintech, Chicago, IL, USA) antibodies, following a standard immunohistochemistry (IHC) protocol. Stained kidneys were examined under a microscope (Leica ICC50 HD, USA), and quantification was performed using the IHC Profiler Plugin in ImageJ Software.

### Statistical analysis

Continuous variables were presented as mean ± standard error of the mean (SEM) or median (25th, 75th percentile), as appropriate. Correlations among continuous variables were assessed using Spearman’s correlation analysis. Categorical variables were expressed as percentages, and differences were evaluated using the chi-square test. Differences in continuous variables between groups were analyzed using Student’s *t*-test or one-way ANOVA, followed by post hoc testing with Tukey’s correction. Statistical analyses were conducted using GraphPad Prism 9.2.0 (GraphPad Software Inc., San Diego, CA, USA), with statistical significance defined as a two-sided *p*-value of < 0.05.

## Results

### HG-induced oxidative stress contributed to PT injury by triggering cell death

**Figure 1.**
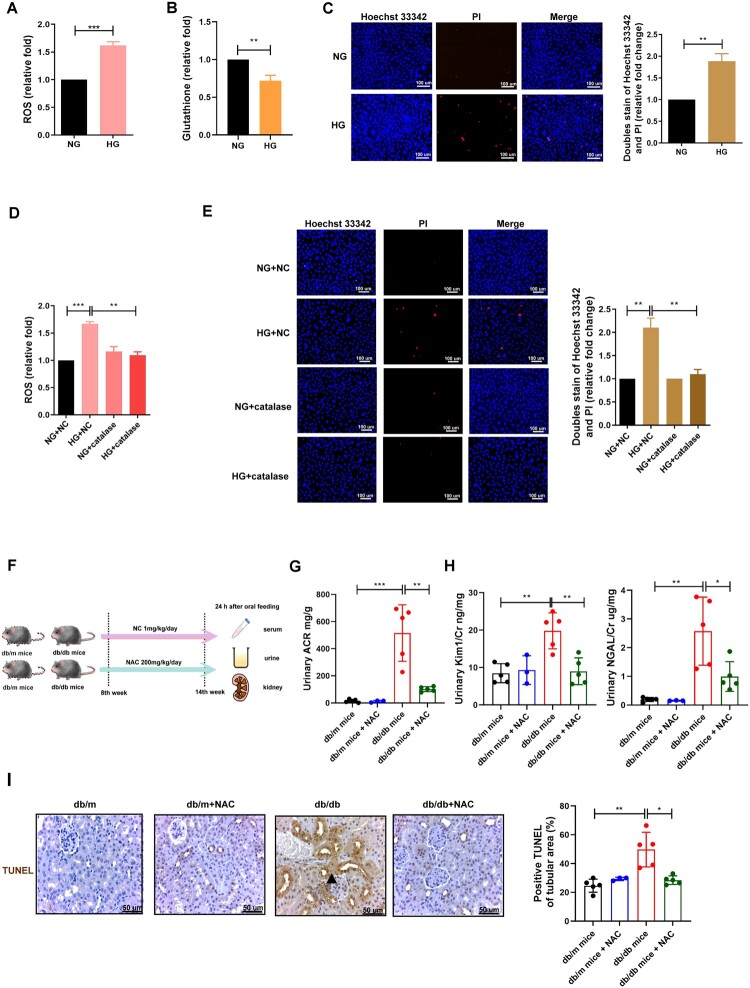
HG-induced oxidative stress contributed PT injury by triggering cell death. (A, B) ROS and glutathione were measured in HK-2 cell treated with NG (5.5 mM) and HG (25 mM) for 48 h using ROS-Glo H_2_O_2_ assay system and glutathione assay respectively. (C) Cell death was measured in HK-2 cell treated with NG and HG for 48 h by Hoechst 33342/PI doubling staining. After pretreatment of catalase (0.4 μM) for 24 h, (D, E) ROS and cell death were examined in HK-2 cell under NG or HG condition for 48 h using ROS-Glo H_2_O_2_ assay system and Hoechst 33342/PI doubling staining. (F) Flow chart of intervention study with NAC in vivo. Blood, urine and kidneys of mice of four groups including db/m mice (*n* = 5), db/m mice + NAC (200 mg/kg/day for 6 weeks, *n* = 3), db/db mice (*n* = 5) and db/db mice + NAC (200 mg/kg/day for 6 weeks, *n* = 5) were collected. (G, H) Urinary albumin/creatinine ratio (ACR), and KIM-1/Cr and NGAL/Cr were assessed in mice. Levels of urinary albumin were assessed using the immunoturbidimetric assay with Tina-quant Albumin Gen.2. Urine creatinine levels were determined using the enzymatic method. Serum creatinine was measured using the compensated Jaffé method. The level of NGAL and KIM-1 in urine was measured using ELISA. (I) TUNEL stain was used to detect cell death in the kidneys of the four groups. Arrows indicated the area of cell death. The images quantification was performed using the IHC Profiler Plugin of ImageJ Software. The bar graph represents the mean ± SEM of at least three independent experiments. *p < 0.05, **p < 0.01, ***p < 0.001 by Student’s *t*-test. Continuous variables were examined using Spearman correlation analysis.

As oxidative stress has been shown to promote the development of CKD [31], we examined the role of oxidative stress in PT injury in DKD. HG increased oxidative stress events, including ROS production, and decreased the levels of the antioxidant glutathione (Figure 1(A,B)), subsequently leading to non-apoptotic cell death in HK-2 cells, as supported by the lack of condensed chromatin in HG-treated HK-2 cells by Hoechst 33342/PI double staining (Figure 1(C)). The antioxidant catalase prevented HG-induced ROS production and cell death (Figure 1(D,E)), suggesting that oxidative stress is involved in HG-mediated cell death in PT. More importantly, in vivo studies (Figure 1(F)) revealed that the administration of antioxidant (NAC) improved kidney injury in db/db mice by decreasing albuminuria (Figure 1(G)), urinary KIM-1/Cr, and NGAL/Cr levels (Figure 1H), and ameliorating cell death in PT detected by TUNEL staining (Figure 1(I)). NAC did not alter body weight or serum glucose levels in db/m and db/db mice (Figure S2(A,B)). These findings indicate that HG induces cell death through oxidative stress in the PT of DKD cells in both in vitro and in vivo models.

### HG-promoted ferroptosis in PT of DKD

**Figure 2.**
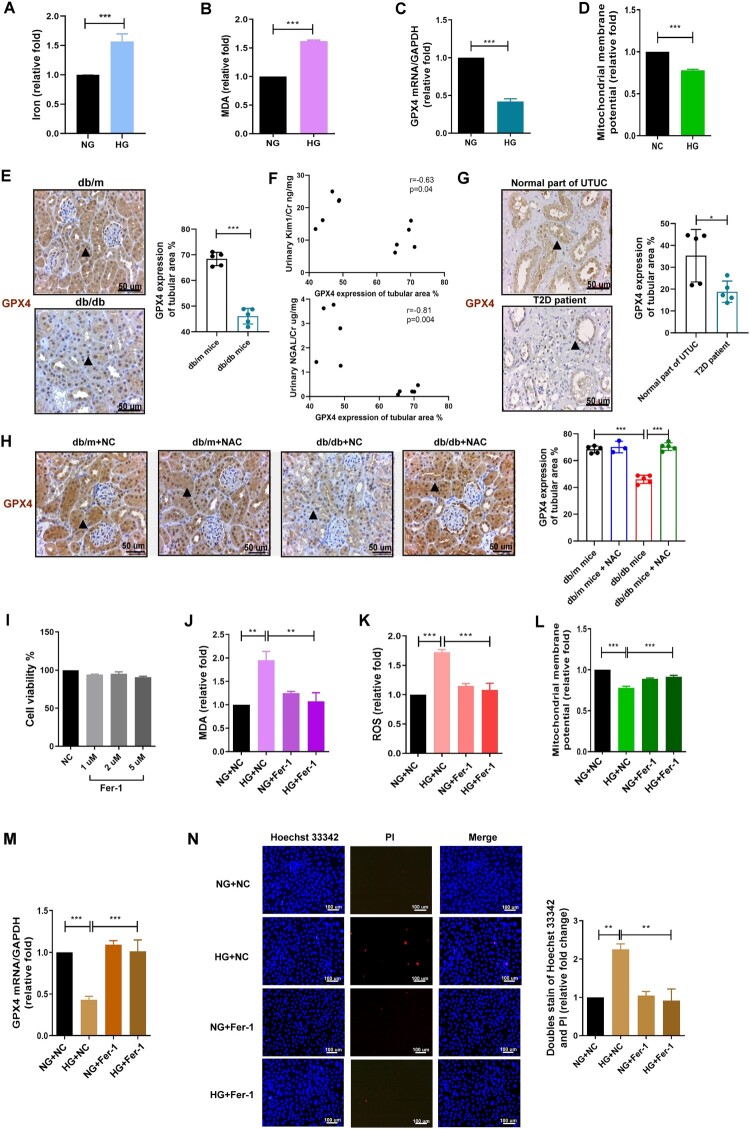
HG promoted ferroptosis in proximal tubule of DKD. HK-2 cells were treated with NG and HG for 48 h. (A, B) Iron and MDA were detected using an iron assay and a lipid peroxidation assay kit. (C)The mRNA level of GPX4 was measured using qRT-PCR. (D) MMP was measured in cells using a rhodamine 123 assay. (E) Expression of GPX4 in the proximal tubules of mouse and human kidneys using IHC staining. Kidney sections from non-diabetic db/m mice and diabetic db/db mice at 14th week were stained with GPX4 (brown). *N* = 5 in each group. Arrows indicate the area of GPX4 expression. (F) Correlation between GPX4 expression in the proximal tubule and urinary KIM-1/Cr and NGAL/Cr in mice at 14th week. Urine creatinine levels were determined by enzymatic methods. The serum creatinine levels were measured using the compensated Jaffé method. The levels of NGAL and KIM-1 in urine were measured using ELISA. (G) Expression of GPX4 in the proximal tubules of human kidneys using IHC staining. Kidney sections of patients with upper tract urothelial carcinoma (UTUC) and T2D were stained with GPX4 (brown). *N* = 5 in each group. Arrows indicate the area of GPX4 expression. (H) The expression of GPX4 in the proximal tubule of kidneys in mice of four groups including db/m mice (*n* = 5), db/m mice + NAC (200 mg/kg/day for 6 weeks, *n* = 3), db/db mice (*n* = 5), and db/db mice + NAC (200 mg/kg/day for 6 weeks, *n* = 5), as measured by IHC staining. Kidney sections were stained with GPX4 (brown). Arrows indicate the area of GPX4 expression. Images were quantified using the IHC Profiler Plugin of ImageJ Software. (I) Viability of HK-2 cells treated with different concentrations of ferrostatin-1 (Fer-1) for 48 h was measured using the WST-1 assay. (J–L) After pretreatment with Fer-1 (2 μM) for 24 h, MDA, ROS, and MMP were examined in Fer-1-treated HK-2 cells under NG or HG conditions for 48 h using a lipid peroxidation assay kit, ROS-Glo H_2_O_2_ assay system, and Rhodamine 123. (M) The mRNA level of GPX4 was measured by qRT-PCR. (N) Cell death was examined by Hoechst 33342/ PI doubling staining. The bar graph represents mean ± SEM of at least three independent experiments. *p < 0.05, **p < 0.01, ***p < 0.001 by Student’s *t*-test. Continuous variables were examined using Spearman’s correlation analysis.

As HG increased oxidative stress and non-apoptotic cell death in PT (Figure 1(A,E)), we evaluated ferroptosis as a type of ROS-dependent cell death triggered in PT by HG. Ferroptosis is characterized by cellular iron accumulation, lipid peroxidation (MDA increase), GPX4 inhibition, and reduced MMP [11]. HG enhanced cellular iron accumulation and MDA production but decreased GPX4 and MMP levels in HK-2 cells (Figure 2(A–D)). In in vivo studies, IHC staining revealed a decreased level of GPX4 in PT area of kidneys of db/db mice (Figure 2(E)). GPX4 levels in PT were negatively correlated with the levels of kidney injury markers, such as urinary KIM1/Cr and NGAL/Cr in mice (Figure 2(F)). We also investigated GPX4 expression in the human kidneys (Table S2). The mean ages of the DKD patients and the UTUC patients were 51.1 ± 11.2 and 61.6 ± 6.5 years-old respectively, and all patients were male. The mean T2D duration was four years. There was no significance in age, sex, and baseline kidney function. Low GPX4 expression levels were found in the PT area of the kidney in T2D patients (Figure 2(G)). In addition, the antioxidant NAC prevented the decrease in GPX4 levels in db/db mice (Figure 2(H)). Fer-1, a ferroptosis inhibitor, reversed MDA and ROS production and MMP loss but without cytotoxicity in HK-2 cells (Figure 2(I–L)). Fer-1 also rescued GPX4 inhibition and cell death in HG-induced PT (Figure 2(M,N)). These findings suggest that HG induces oxidative stress and further promotes ferroptosis in PT, and that ferroptosis plays a pathophysiological role in DKD.

### HG enhanced ferroptosis through suppressing GCLM expression in DKD

**Figure 3.**
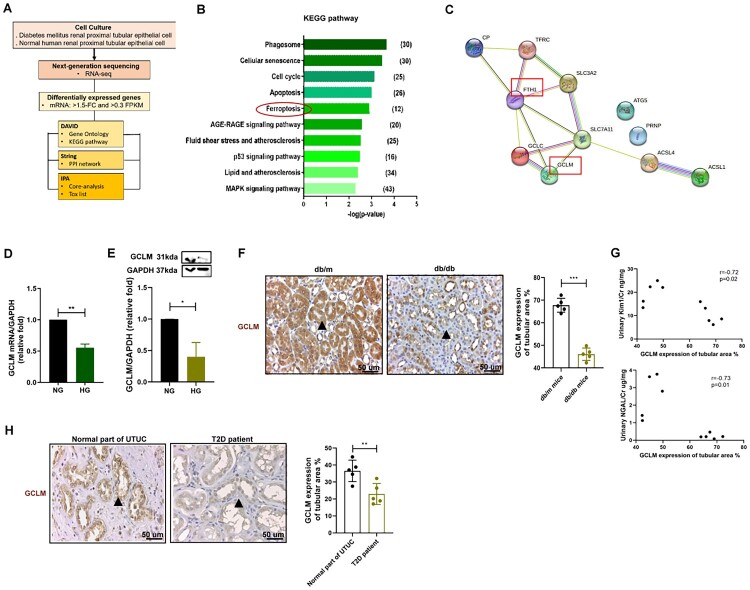
HG promoted ferroptosis through suppressing GCLM expression in DKD. (A) Flowchart of identification of potential mRNAs related to ferroptosis from renal PTECs (RPTECs) of a normal individual and a type 2 diabetic patient. (B) Top 10 KEGG pathway of RPTECs in the type 2 diabetic patient compared to the normal individual. (C) The protein-protein interaction network analysis of genes associated ferroptosis. STRING database (version 10) was used in bioinformatic analysis. (D, E)The mRNA and protein levels of GCLM in HK-2 cell treated with NG or HG for 48 h were measured by qRT-PCR and western blotting. (F) The expression GCLM in proximal tubule of mice kidneys. Kidney sections of non-diabetic db/m mice and diabetic db/db mice at 14th week were stained with GPX4 (brown). *N* = 5 in each group. Arrows indicated the area of GCLM expression. The images quantification was performed using the IHC Profiler Plugin of ImageJ Software. (G) The correlation between GCLM expression in proximal tubule and urinary KIM-1/Cr and NGAL/Cr in mice at 14th week. Urine creatinine levels were determined using the enzymatic method. Serum creatinine was measured using the compensated Jaffé method. The level of NGAL and KIM-1 in urine was measured using ELISA. (H) The expression GCLM in proximal tubule of human kidneys. Kidney sections of upper tract urothelial carcinoma (UTUC) patients, and T2D patients were stained with GPX4 (brown). *N* = 5 in each group. Arrows indicated the area of GCLM expression. The bar graph represents the mean ± SEM of at least three independent experiments. RPM, read per million. *p < 0.05, **p < 0.01, ***p < 0.001 by Student’s *t*-test. Continuous variables were examined using Spearman correlation analysis.

To further explore the potential molecular mechanisms contributing to ferroptosis, bulk RNA sequencing and bioinformatics analyses were used to analyze the transcriptome from primary PTECs of the T2D patient and the normal individual (Figure 3(A)). Of the 1541 mRNAs with a 1.5-fold change in PTECs from the T2D patient compared to those from the normal individual, 865 were downregulated and 676 were upregulated. KEGG pathway analysis revealed that ferroptosis was one of the top five pathways (Figure 3(B)), while the public GEO database, GSE86300 was additionally used to validate our results [25]. GSE86300 revealed that db/db mice had lower GCLM expression levels and GCLM was involved in ferroptosis, which was one of the top 10 KEGG pathways of downregulated hub genes in db/db mice compared to db/m mice (Table S3) [25]. Among the mRNAs predicted to be involved in ferroptosis (Table 1), GCLM had a strong interaction with the ferroxidase enzyme ferritin heavy chain 1 (FTH1) (Figure 3(C)). Bulk RNA sequencing revealed decreased GCLM mRNA expression in the T2D patient compared to that in the normal individual (Table S4). HG decreased the expression of GCLM in HK-2 cells at both the mRNA and protein level (Figure 3(D,E)). IHC staining revealed lower levels of GCLM in the PT of db/db mice (Figure 3(F)); additionally, and the expression level of GCLM was negatively correlated with kidney injury markers, including urinary KIM-1/Cr and NGAL/Cr in the mice (Figure 3(G)). GCLM expression levels were decreased in T2D patients (Figure 3(H)). These results suggest that GCLM participates in the progression of DKD through ferroptosis and oxidative stress.

**Table 1.** Top five KEGG pathway of PTECs between T2D and normal individuals based on DAVID database.

KEGG pathway	*p*-value	Genes
Phagosome	0.0002	ITGB1, RAB5C, TFRC, THBS1, THBS4, ACTB, ACTG1, C3, COMP, TUBA1B, TUBB6, HLA-DMA, HLA-DMB, TUBA1A, TUBB3, ATP6V0A4, RAC1, HLA-DOA, RILP, ITGA2, TUBB, M6PR, TAP2, HLA-C, HLA-A, TUBB4B, TUBA4A, CANX, HLA-DRB1, RAB7A
Cellular senescence	0.0003	ITPR1, LIN9, FOXO1, PPP3CB, CCNB1, CCND2, MYC, CHEK1, E2F1, E2F2, MYBL2, CAPN1, HRAS, SMAD3, GADD45A, RRAS2, HLA-C, TSC1, HLA-A, SIRT1, CDC25A, TGFBR2, PPP1CA, MRAS, CCNE1, MDM2, CALM3, CALM1, SQSTM1, RAD9A
Cell cycle	0.0007	CDKN1C, ANAPC15, PCNA, PRKDC, ANAPC10, FZR1, CCNB1, CCND2, ORC1, PTTG1, MYC, ORC3, CHEK1, E2F1, E2F2, SFN, CDKN2C, SMAD3, GADD45A, CDC6, YWHAZ, CDC25A, CCNE1, MDM2, MCM4
Apoptosis	0.0010	ITPR1, ACTB, ACTG1, LMNB1, TUBA1B, MAPK8, CASP8, BCL2L11, TUBA1A, TNFSF10, CAPN1, BID, HRAS, CTSD, SPTAN1, CTSB, APAF1, GADD45A, PARP2, TRAF2, CFLAR, NGF, TUBA4A, TNFRSF1A, ERN1, ATF4
Ferroptosis	0.0012	PRNP, GCLC, TFRC, ACSL1, FTH1, SAT2, ACSL4, SLC3A2, SLC7A11, **GCLM**, CP, ATG5

Note: Bold value reveals the target gene ‘GCLM’ involved in ferroptosis KEGG pathway.

**Figure 4.**
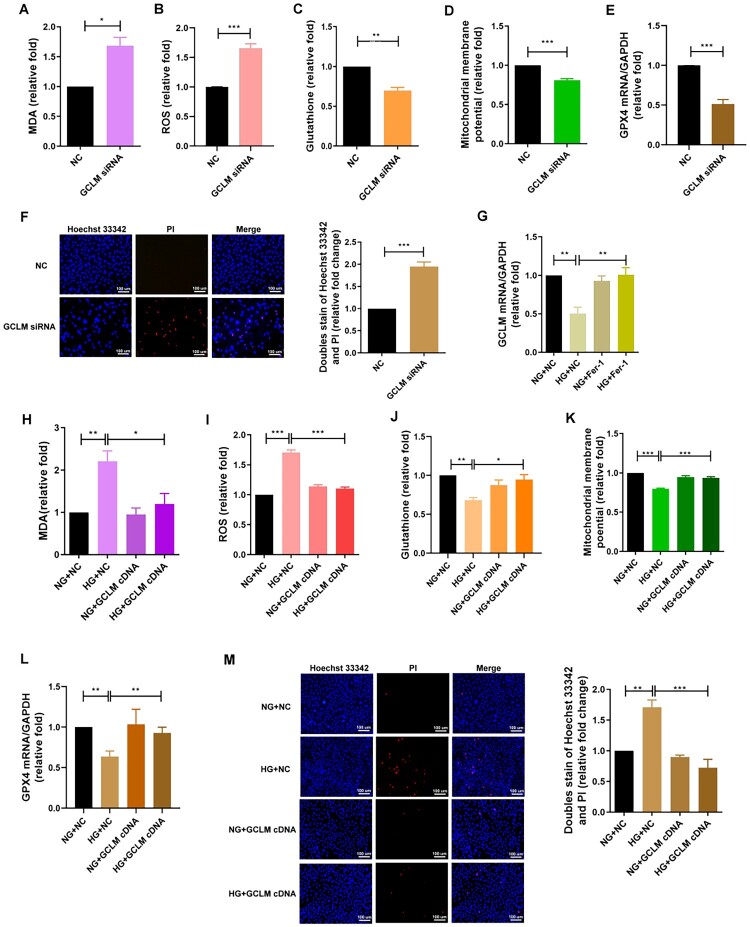
Loss-and gain-of-function of GCLM in ferroptosis of DKD. HK-2 cells were transfected with GCLM siRNA (20 nM) or normal control (NC, 20 nM), and 24 h after transfection, the cells were treated with NG for 48 h. HK-2 cells were transfected with GCLM cDNA or control plasmid, and 24 h after transfection, the cells were treated with NG or HG for 48 h. (A, H) MDA (B, I) ROS (C, J) glutathione (D, K) MMP and (E, L) GPX4 mRNA levels were measured in cells using the lipid peroxidation assay kit, ROS-Glo H_2_O_2_ assay system, glutathione assay, rhodamine 123 assay, and qRT-PCR. (F, M) Cell death was measured in GCLM siRNA- or cDNA-transfected HK-2 cells treated with NG or HG for 48 h by Hoechst 33342/PI double staining. (G) After pretreatment with Fer-1 (2 μM) for 24 h, the mRNA levels of GCLM in HK-2 cells treated with NG or HG for 48 h were measured by qRT-PCR. The bar graph represents mean ± SEM of at least three independent experiments. *p < 0.05, **p < 0.01, ***p < 0.001 by Student’s *t*-test or ANOVA followed by the post hoc test with Tukey’s correction.

The role of GCLM in ferroptosis in DKD was examined using loss-and-gain-of-function models. Silencing of GCLM by siRNA transfection and overexpression of GCLM by cDNA transfection were performed in HK-2 cells by qRT-PCR and western blotting (Figure S3(A,B)). Silencing GCLM increased MDA and ROS levels (Figure 4(A,B)) and decreased glutathione production, MMP, and GPX4 mRNA expression (Figure 4(C–E)). Suppression of GCLM expression resulted in HK-2 cell death (Figure 4(F)), whereas pretreatment with Fer-1 reversed HG-induced GCLM suppression (Figure 4(G)). Conversely, overexpression of GCLM ameliorated HG-promoted ferroptosis features, including increased MDA and ROS production (Figure 4(H,I)) and decreased expression of glutathione, MMP, and GPX4 mRNA (Figure 4(J–L)). Ectopic expression of GCLM also rescued HK-2 cell death under HG conditions (Figure 4(M)). HG promoted ferroptosis in HK-2 cells by suppressing GCLM.

### SGLT2i treatment ameliorated ferroptosis-induced PT injury through modulating GCLM in DKD

**Figure 5.**
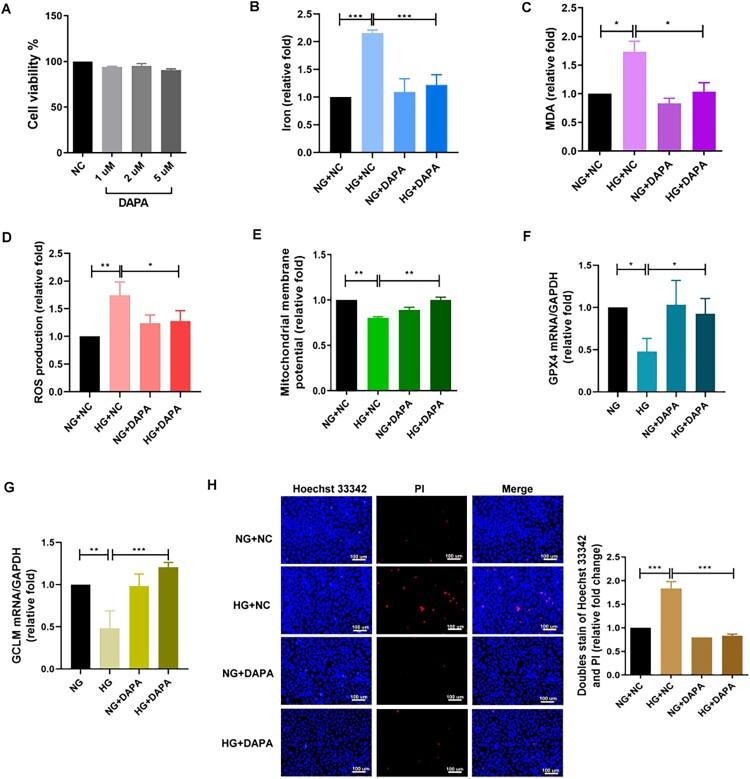
SGLT2i ameliorated ferroptosis-induced proximal tubular injury through modulating GCLM in DKD in vitro. (A) Cell viability was assessed in HK-2 cell treated with different concentrations of dapagliflozin (DAPA). After pretreatment of DAPA (2 μM) for 24 h, (B–E) Iron, MDA, ROS, and MMP were measured in DAPA-treated HK-2 cell under NG or HG condition for 48 h using iron assay kit, lipid peroxidation assay kit, ROS-Glo H_2_O_2_ Assay system, and rhodamine 123 assay respectively. (F, G) After pretreatment of DAPA for 24 h, the mRNA levels of GPX4 and GCLM in HK-2 cell treated with NG or HG for 48 h were measured by qRT-PCR. (H) Cell death was measured in cells by Hoechst 33342/PI doubling staining. *p < 0.05, **p < 0.01, ***p < 0.001 by Student’s *t*-test or ANOVA followed by the post hoc test with Tukey’s correction.

Because of the strong therapeutic effect of SGLT2i on DKD, we treated HK-2 cells with the SGLT2i dapagliflozin (DAPA, 2 μM) under NG and HG conditions (Figure 5(A)) and found that dapagliflozin reversed HG-promoted ferroptosis triggers, including cellular iron accumulation, MDA production and ROS generation, MMP loss, and GPX4 downregulation in HK-2 cells (Figure 5(B–F)). In addition, GSE185801 revealed that SGLT2i reversed the decreased GCLM expression in db/db mice (Table S5) [26]. Dapagliflozin could ameliorated HG-induced suppression of GCLM in HK-2 cells (Figure 5(G)). Moreover, dapagliflozin ameliorated HG-induced cell death in HK-2 cells (Figure 5(H)).

**Figure 6.**
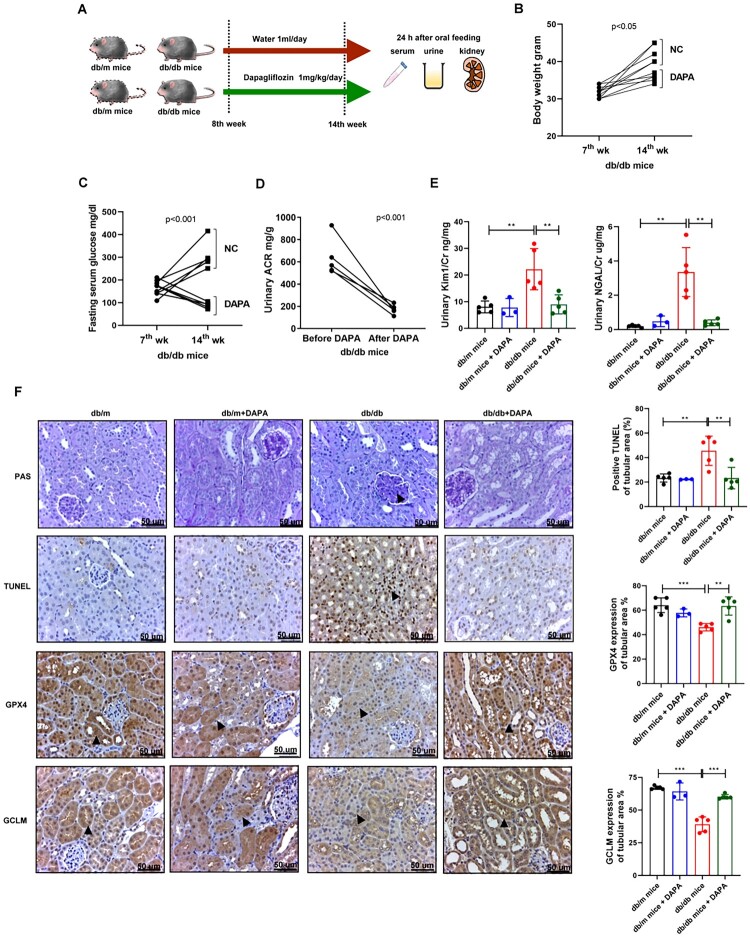
SGLT2i ameliorated ferroptosis-induced proximal tubular injury through modulating GCLM in DKD in vivo. (A) Flow chart of intervention study with dapagliflozin (DAPA) in in vivo. (B, C) Body weight and blood sugar were shown in mice during experiment period. (D) Urinary albumin/creatinine (ACR) was decreased after DAPA treatment in db/db mice. (E) Urinary KIM-1/Cr and NGAL/Cr levels were assessed in mice of four groups including db/m mice (*n *= 5), db/m mice + DAPA (dapagliflozin 1 mg/kg/day for 6 weeks, *n* = 3), db/db mice (*n* = 5) and db/db mice + DAPA (*n* = 5). Urine creatinine levels were determined using the enzymatic method. Serum creatinine was measured using the compensated Jaffé method. Levels of urinary albumin were assessed using the immunoturbidimetric assay with Tina-quant Albumin Gen.2. The level of NGAL and KIM-1 in urine was measured using ELISA. (F) Periodic acid-Schiff (PAS) stain and TUNEL stain of kidneys in mice. Arrows indicated the area of mesangial matrix expansion in PAS statin and cell death in TUNEL stain. The expressions of GPX4 and GCLM in proximal tubule of kidneys in mice. The kidney sections were stained with GPX4 and GCLM (brown). Arrows indicated the area of GPX4 or GCLM expression. The images quantification was performed using the IHC Profiler Plugin of ImageJ Software. The bar graph represents the mean ± SEM of at least three independent experiments. *p < 0.05, **p < 0.01, ***p < 0.001 by Student’s *t*-test or ANOVA followed by the post hoc test with Tukey’s correction.

Further in vivo studies were performed to investigate the role of SGLT2i in ferroptosis in DKD. We treated 8-week-old db/db mice with dapagliflozin 1 mg/kg/day for 6 weeks. The mice were sacrificed at 14th week, and blood, urine, and kidney specimens were collected (Figure 6(A)). After dapagliflozin treatment, body weight and fasting sugar levels were lower in db/db mice than in those treated with NC (Figure 6(B,C)). Dapagliflozin diminished urinary albuminuria (Figure 6(D)), KIM-1/Cr and NGAL/Cr levels in db/db mice (Figure 6(E)). Furthermore, TUNEL staining revealed that dapagliflozin ameliorated cell death in the PT of db/db mice (Figure 6(F)). IHC staining showed that dapagliflozin reversed the decreased levels of GPX4 and GCLM in the PT of db/db mice (Figure 6(F)). These findings indicate that SGLT2i treatment rescues DKD-induced ferroptosis in PT by modulating GCLM expression.

## Discussion

**Figure 7.**
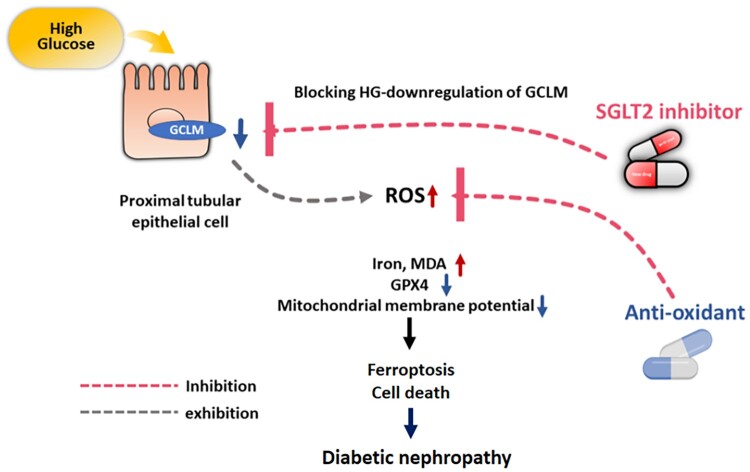
Illustration of the mechanism of HG-induced ferroptosis by suppressing GCLM expression and SGLT2i reduced HG-induced ferroptosis through upregulation of GCLM expression in DKD.

This study demonstrates that ferroptosis participates in the pathophysiology of DKD in both in vitro and in vivo models. HG-induced ROS production, iron accumulation, and lipid peroxidation and decreased GPX4 levels and MMP, subsequently leading to ferroptotic cell death in PT by suppressing GCLM expression. Furthermore, treatment with SGLT2i improved DKD progression by ameliorating GCLM-mediated ferroptosis. Our results provide novel insights into the potential impact of ferroptosis on DKD progression and the molecular mechanisms of SGLT2i in DKD (Figure 7).

Programmed cell death plays an important role in the onset and progression of DKD [32]. For example, podocyte apoptosis results in podocyte depletion and glomerular injury, leading to albuminuria and glomerular structure damage [33]. Apoptosis in PT cells results in tubular atrophy with loss of kidney function [34], and necroptosis participates in the pathogenesis of DKD via the ubiquitin C-terminal hydrolase L1 (UCHL1)-RIPK1/RIPK3 pathway [35]. Ferroptosis is an iron-dependent form of programmed cell death, and accumulating evidence has revealed that it mediates acute kidney injury [36]. Ferroptosis can be inhibited by the enzymatic activity of the two key antioxidant systems. The first is GPX4, which catalyzes the reduction of lipid peroxides in a glutathione-dependent reaction. The second is the ferroptosis suppressor protein, which facilitates ubiquinone regeneration and serves as a trap for lipid peroxyl radicals. The therapeutic effects of antioxidants in ferroptosis-mediated DKD remain unclear. A few studies have reported that antioxidants, including Fer-1 and NAC, can prevent ferroptosis in DKD [37,38]. Tang et al. reported that NAC could maintain mitochondrial redox homeostasis to alleviate ferroptosis in DKD [37]. Zheng et al. reported that Fer-1 treatment reduces the expression of ZRT/IRT-like protein 14 and the levels of ferrous iron and MDA in DKD [38]. Our findings reveal that HG induces ferroptotic cell death in an ROS-dependent manner. Furthermore, scavenging by NAC prevented PT injury and DKD progression in vivo, providing a new therapeutic strategy for DKD.

Nevertheless, the pathophysiological role of ferroptosis and its signal transmission in DKD has not been well explored. Our results show that HG decreases the anti-ferroptosis marker (GPX4) by suppressing GCLM expression to promote PT injury. GCLM is a rate-limiting enzyme involved in the synthesis of glutathione, which is essential for cellular redox homeostasis and antioxidant defense [39], and is regulated by nuclear factor erythroid 2–related factor 2 (NRF2) and an important endogenous antioxidant. GCLM depletion has been associated with mitochondrial dysfunction, lung inflammation, and ovarian failure [40]. Nevertheless, it remains unclear whether HG regulates GCLM to induce PT injury through ferroptosis in DKD. Our study indicates that HG induces GCLM downregulation, which in turn triggers oxidative stress and results in ferroptotic cell death. The critical role of GCLM is supported by the downregulation of GCLM, resulting in ROS production and lipid oxidation, leading to PT injury, whereas the upregulation of GCLM rescued PT cells from oxidative stress and ferroptotic cell death. Moreover, GCLM loss is strongly associated with kidney injury. In summary, our results confirm the pathophysiological role of GCLM in DKD and show that GCLM deficiency contributes to ferroptosis and subsequent DKD progression.

Some studies have identified potential mechanisms that regulate GCLM expression. Park et al. found that honokiol increased GCLM by activating NRF2 via phosphatidylinositol 3-kinase (PI3 K)/protein kinase B (AKT) and protein kinase C (PKC) signaling in renal ischemic and reperfusion injury [41], whereas Gou et al. reported that andrographolide inhibited TNF-α-induced inflammation by upregulating GCLM expression through the PI3 K/AKT/NRF2 and PI3 K/AKT/activating protein-1 (AP-1) pathways [42]. For DKD, chitooligosaccharides increased NRF2 and downstream target gene GCLM expression and substantially decreased Kelch-like ECH-associated protein 1(KEAP1) expression in mouse model [43]. High mobility group box 1 protein (HMGB1) regulated HG-induced ferroptosis through NRF2 pathway and GCLM in mesangial cells [44]. Cyanidin 3-glucoside increases glutathione and GCLM levels and decreases GSSG levels in the kidneys of diabetic mice [45].

In T2D patients with poor sugar control, an increase in glucose filtration results in enhanced sodium-coupled glucose reabsorption by PT and reduced sodium delivery to the macula densa. This further promotes intrarenal activation of the renin–angiotensin–aldosterone system, efferent arteriolar vasoconstriction, glomerular hypertension, and hyperfiltration, which are all important features of the initial stage of DKD [13]. SGLT2i can inhibit alterations in tubuloglomerular feedback, tubule hypertrophy, hypoxia, inflammation, and even fibrosis [13]. SGLT2i not only reduce oxygen requirements and improve cortical tissue oxygenation [13], but also ameliorate the expression of circulating inflammatory molecules, such as interleukin-6 (IL-6) and tumor necrosis factor alpha (TNF-α) [46,47]. SGLT2i have been shown to protect against cardiovascular and kidney complications mediated by suppressing ferroptosis [48–51]. Quagliariello et al. found that empagliflozin reduced cardiac fibrosis and improved myocardial strain in non-diabetic mice treated with doxorubicin [48]. Wu et al. reported that empagliflozin inhibited ferroptosis to promote revascularization in hindlimb ischemia in a diabetes model [49]. Zhang et al. reported that empagliflozin attenuates tubular ferroptosis in DKD through the AMP-activated protein kinase (AMPK)/NRF2 pathway [50], and another study demonstrated that dapagliflozin could ameliorate tubular ferroptosis by stabilizing SLC40A1 [51]. Our findings demonstrate that dapagliflozin reduces ferroptosis by modulating the expression of GCLM in the PT of DKD. However, whether SGLT2i regulated GCLM expression to ameliorate ferroptosis-induced DKD is not well known. Few indirect evidences of potential mechanisms mentioned that SGLT2i enhances NRF2 activation, and then promotes GSH synthesis and GCLM expression through AMPK pathway [52,53]. Further studies are necessary to determine whether SGLT2i upregulates GCLM expression to ameliorate ferroptosis and rescue DKD progression.

The current study used multi-disciplinary methods, including bulk RNA sequencing, in vitro and in vivo models, and human kidney samples, to determine the molecular mechanism of SGLT2i in ferroptosis-mediated DKD. However, our study has some limitations. We did not silence or overexpress GCLM expression in the mice and did not isolate PT from the mice to validate the results of in vitro model. In addition, the signaling pathway of SGLT2i regulating GCLM is needed to explore in the future. Finally, owing to the transcriptomic analysis including only one sample per group, we utilized GEO databases (GSE86300) to increase credibility of bulk RNA-seq analysis [25]. SGLT2i have been considered as a principle therapeutic agent for DKD based on American Diabetes Association (ADA) and Kidney Disease Improving Global Outcomes (KDIGO) guidelines [54,55]. In real world, only few patients who received SGLT2i treatment need kidney biopsies for unknown cause of rapid decline in kidney function. Kidney samples from T2D patients taking SGLT2i were limited. Thus, GEO database (GSE185801) was used to validate our clinical results [26].

In conclusion, our findings demonstrate that HG promotes oxidative stress and the consequent ferroptosis by modulating GCLM in DKD. SGLT2i treatment ameliorates oxidative stress and ferroptosis in DKD by enhancing GCLM expression. GCLM has the potential as a protective agent against DKD and the therapeutic mechanism of SGLT2i in DKD.

## Supplementary Material

Author Checklist.pdf

supplemental materials_1140622.docx

## Data Availability

The data of transcriptomic analysis in the present study can be found in the GEO database under accession numbers (GSE86300, GSE185801, and GSE185586).
