# Supplementary material for: Sodium–glucose cotransporter 2 inhibitors ameliorate glutathione cysteine ligase modifier-mediated oxidative stress and subsequent ferroptosis in proximal tubules of diabetic kidney disease
Source: Redox Rep. 2025 Jul 28;30(1):2528334. doi: 10.1080/13510002.2025.2528334 (PMC12308872; doi:10.1080/13510002.2025.2528334)

**Table S1.** The sequence of GCLM cDNA and siRNA, and NC cDNA and siRNA.

|  | **NC cDNA** |
| --- | --- |
|  | EX-NEG-M92 Datasheet-control |
|  | Suggested Sequencing Primers: |
|  | Forward: 5'-CAGCCTCCGGACTCTAGC-3' |
|  | Reverse: 5'-CTCTACAAATGTGGTATGGC-3' |
|  | **GCLM cDNA** |
|  | Homo sapiens GCLM, transcript variant 1, mRNA. |
|  | Suggested Sequencing Primers: |
|  | Forward: 5'-CAGCCTCCGGACTCTAGC-3' |
|  | Reverse: 5'-CTCTACAAATGTGGTATGGC-3' |
|  |  |
|  | **NC siRNA** |
|  | **ON-TARGET Non-targeting** |
| **Target Sequence** | UGGUUUACAUGUCGACUAA |
| **Target Sequence** | UGGUUUACAUGUUGUGUGA |
| **Target Sequence** | UGGUUUACAUGUUUUCUGA |
| **Target Sequence** | UGGUUUACAUGUUUUCCUA |
|  |  |
|  | **GCLM siRNA** |
|  | ON-TARGET siRNA J-011670-09 |
| **Target Sequence** | CAGAUAUUCUUUCGUCAUA |
|  | ON-TARGET siRNA J-011670-10 |
| **Target Sequence** | GGUAUUCGGUCAUUGUGAA |
|  | ON-TARGET siRNA J-011670-11 |
| **Target Sequence** | UAUCAGUGGGCACAGGUAA |
|  | ON-TARGET siRNA J-011670-12 |
| **Target Sequence** | GAAUGAAUGGAUUCCCAA |

**Table S2.** **The characteristics parameters in human participants**

|  | UTUC  N = 5 | Type 2 Diabetes  N = 5 | *P*-value |
| --- | --- | --- | --- |
| Age, years | 61.6 ± 6.5 | 51.1 ± 11.2 | 0.14 |
| Sex (male), % | 100% | 100% | - |
| T2D duration, years | - | 4.0 ± 7.1 | <0.001 |
| Blood urea nitrogen, mg/dL | 16.1 ± 4.0 | 21.9 ± 5.3 | 0.14 |
| Serum creatinine, mg/dL | 1.2 ± 0.3 | 1.7 ± 0.8 | 0.24 |
| Estimated glomerular filtration rate, mL/min/1.73m^2^ | 68.4 ± 20.9 | 55.4 ± 28.9 | 0.49 |
| Glycated hemoglobulin, % | - | 7.0 ± 0.2 | <0.001 |
| Urine protein/creatinine ratio, mg/g | < 150 | 9216.8 ± 5251.9 | <0.001 |

**Table S3.** **KEGG pathway of down-regulated hub genes (db/db *v.s.* db/m) in GSE86300**

| **Term** | **Count** | **Genes** |  |
| --- | --- | --- | --- |
| Metabolic pathways | 51 | PNLIPRP1, ACSM3, PANK1, CSAD, AACS, PPCDC, GYS2, HYKK, UGT2A3, CSGALNACT1, GPX6, DHDH, BDH1, AGPS, RDH5, MVD, IDO2, BCAT1, DGKH, B4GALT5, PDE1A, ODC1, ALOX15, AK5, HSD17B11, DDO, HSD11B1, ALDH3B2, NT5E, CBS, HSD17B1, AFMID, LDHD, ESD, HSD17B2, ASPDH, PDE6A, B3GALNT2, SRD5A2, PDE6G, ST8SIA1, GLYCTK, FMO3, FMO5, GCLC, P4HA1, PI4KA, CYCS, PPT2, DEGS2, GCLM |  |
| Steroid hormone biosynthesis | 7 | HSD11B1, SRD5A2, HSD17B1, HSD17B2, UGT2A3, HSD17B11, CYP7B1 |  |
| Biosynthesis of cofactors | 10 | GCLC, PANK1, AFMID, ASPDH, UGT2A3, AK5, IDO2, BCAT1, GCLM, PPCDC |  |
| Hypertrophic cardiomyopathy | 8 | RYR2, ACE, ACTC1, LAMA2, ITGA10, TNNC1, TNNI3, ITGB6 |  |
| Pantothenate and CoA biosynthesis | 4 | PANK1, CSAD, BCAT1, PPCDC |  |
| cAMP signaling pathway | 11 | POPDC3, RYR2, HCAR1, SUCNR1, BDNF, PPP1R1B, NPPA, BRAF, TNNI3, ADRB2, KCNK2 |  |
| Dilated cardiomyopathy | | 7 | RYR2, ACTC1, LAMA2, ITGA10, TNNC1, TNNI3, ITGB6 |
| Complement and coagulation cascades | | 6 | CFD, C8G, SERPINF2, MASP2, C8A, CD55 |
| ECM-receptor interaction | | 6 | LAMA2, LAMC3, ITGA10, COL4A4, CD36, ITGB6 |
| Taurine and hypotaurine metabolism | | 3 | FMO3, CSAD, FMO5 |
| **Ferroptosis** | | 4 | GCLC, ALOX15, SLC39A8, **GCLM** |

**Table S4**. GCLC and GCLM mRNA expression levels in PTECs of the normal individual and the T2D patient.

| **Gene Name** | **Diabetic PTEC** | **Normal PTEC** | **Fold change (FC)** | **Log2FC(diabetes/normal)** |
| --- | --- | --- | --- | --- |
| GCLM | 5.00237 | 7.67249 | 0.651987816 | -0.61708 |

**Table S5.** **KEGG pathway of down-regulated hub genes (db/db *v.s.* db/m) and up-regulated hub genes (db/db +dapa *v.s.* db/db) in GSE185801**

| **Term** | **Count** | **Genes** |
| --- | --- | --- |
| Protein processing in endoplasmic reticulum | 18 | HSPA8, XBP1, TRAM1, HSP90AA1, HSP90AB1, DERL3, HSPA4L, DERL2, UBE2G2, DNAJA1, DNAJB1, HSPH1, OSTC, ERP29, CRYAB, HSPA1B, HSPA1A, ATF4 |
| p53 signaling pathway | 9 | CCNB2, STEAP3, CCNB1, CDK1, SHISA5, PMAIP1, TNFRSF10B, CYCS, SFN |
| Cell cycle | 13 | TGFB2, CDCA5, MAD2L1BP, ESCO2, PKMYT1, NDC80, CCNB2, CCNB1, ESPL1, E2F1, CDK1, SFN, TRIP13 |
| Peroxisome | 8 | AMACR, PRDX1, MPV17L, AGPS, NUDT19, CROT, SLC27A2, SOD1 |
| Apoptosis | 10 | TUBA1C, ENDOG, TUBA1A, PMAIP1, TNFRSF10B, BIRC5, CYCS, NGF, HRAS, ATF4 |
| Butanoate metabolism | 4 | HMGCS1, ACSM3, OXCT1, AACS |
| Complement and coagulation cascades | 7 | C8G, SERPINF2, CFI, PLAUR, CLU, F3, CD55 |
| MAPK signaling pathway | 16 | HSPA8, TGFB2, CSF1, PDGFB, VEGFC, HSPB1, NGF, DUSP8, PDGFD, FGF18, STMN1, HRAS, HSPA1B, HSPA1A, EPHA2, ATF4 |
| Amino sugar and nucleotide sugar metabolism | 5 | GMPPB, CYB5R1, CMAS, CYB5R3, UAP1 |
| Sphingolipid metabolism | 5 | UGCG, CERS5, GLB1, SPHK1, B4GALT5 |
| Taurine and hypotaurine metabolism | 3 | CSAD, GGT1, FMO5 |
| Metabolism of xenobiotics by cytochrome P450 | 6 | HSD11B1, ALDH3B2, UGT2A3, CYP1B1, CYP2E1, ADH7 |
| **Ferroptosis** | 4 | STEAP3, MAP1LC3A, **GCLM**, CP |
| Histidine metabolism | 3 | ALDH3B2, AOC1, CNDP2 |
| Lipid and atherosclerosis | 11 | HSPA8, XBP1, HSP90AA1, HSP90AB1, TNFRSF10B, CYCS, CD36, HRAS, HSPA1B, ATF4, HSPA1A |

**Figure S1.** The cell viability of HK-2 cells treated with different doses of Ferrostatindose-dependent


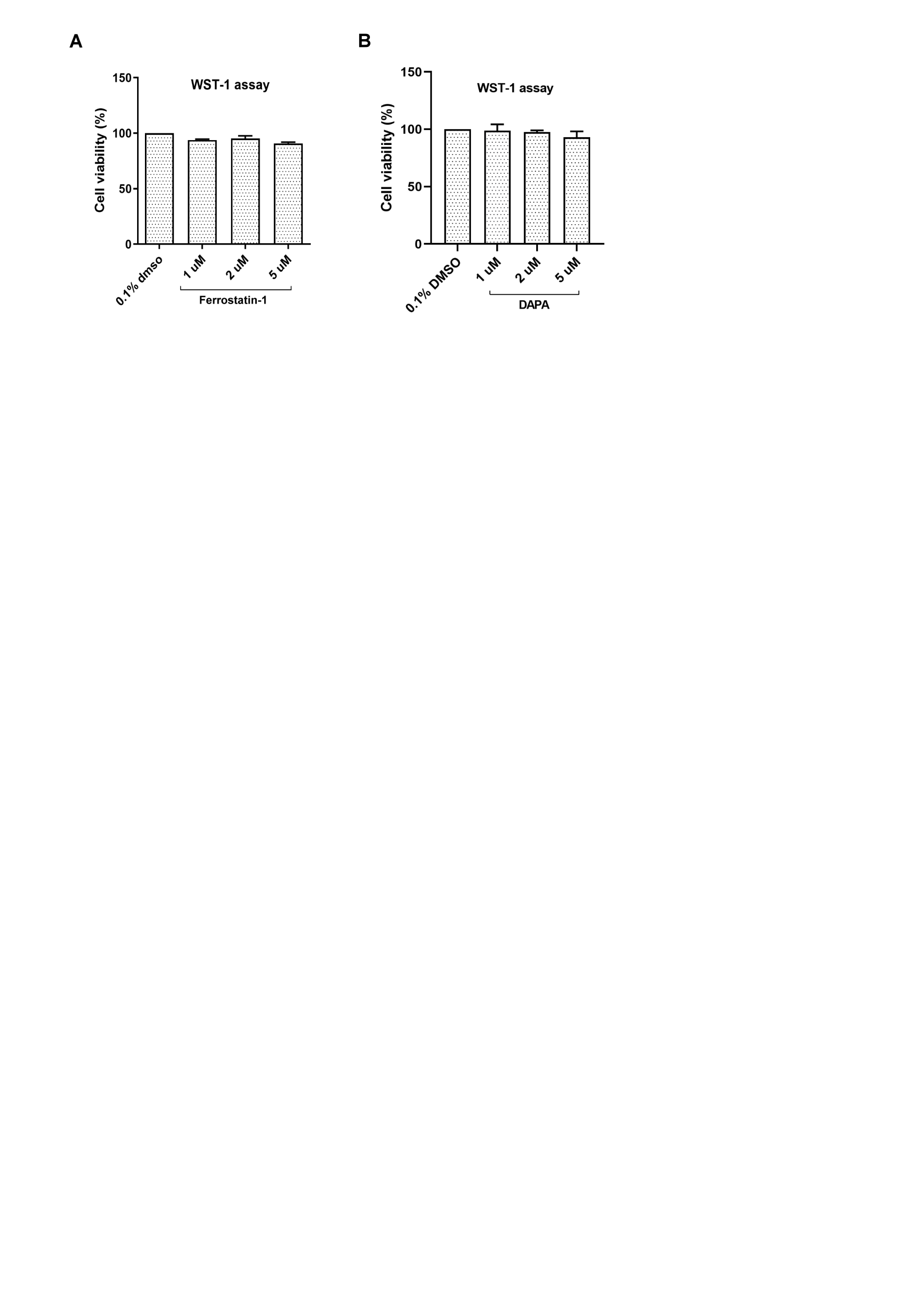


**Figure S2.** The biochemistry data of db/m and db/db mice treated with NAC.

(A, B) Body weight and blood sugar were shown in mice during experiment period


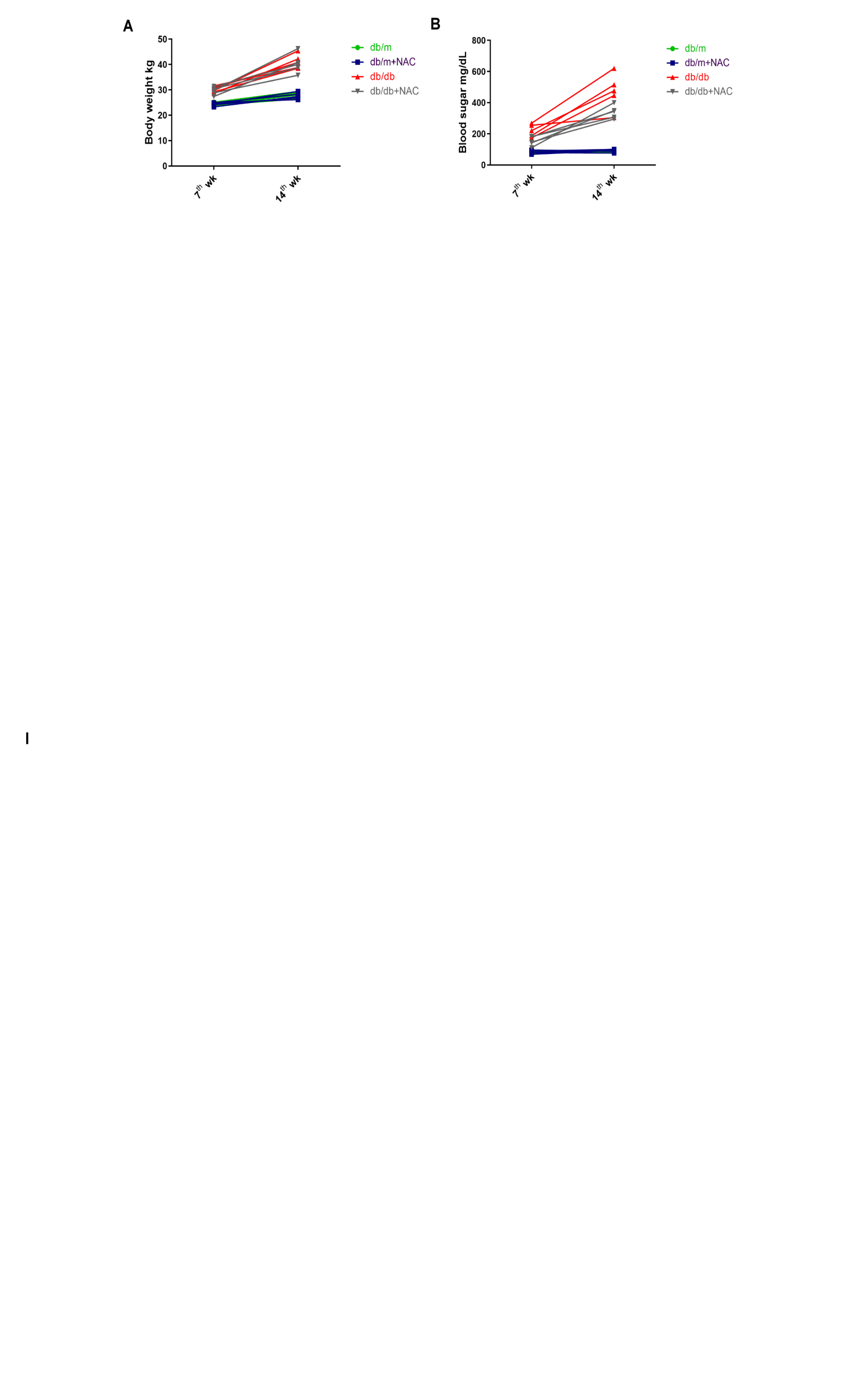


**Figure S3.** The efficiency of suppression and overexpression of GCLM in HK-2 cell measured by qRT-PCR and western blot.


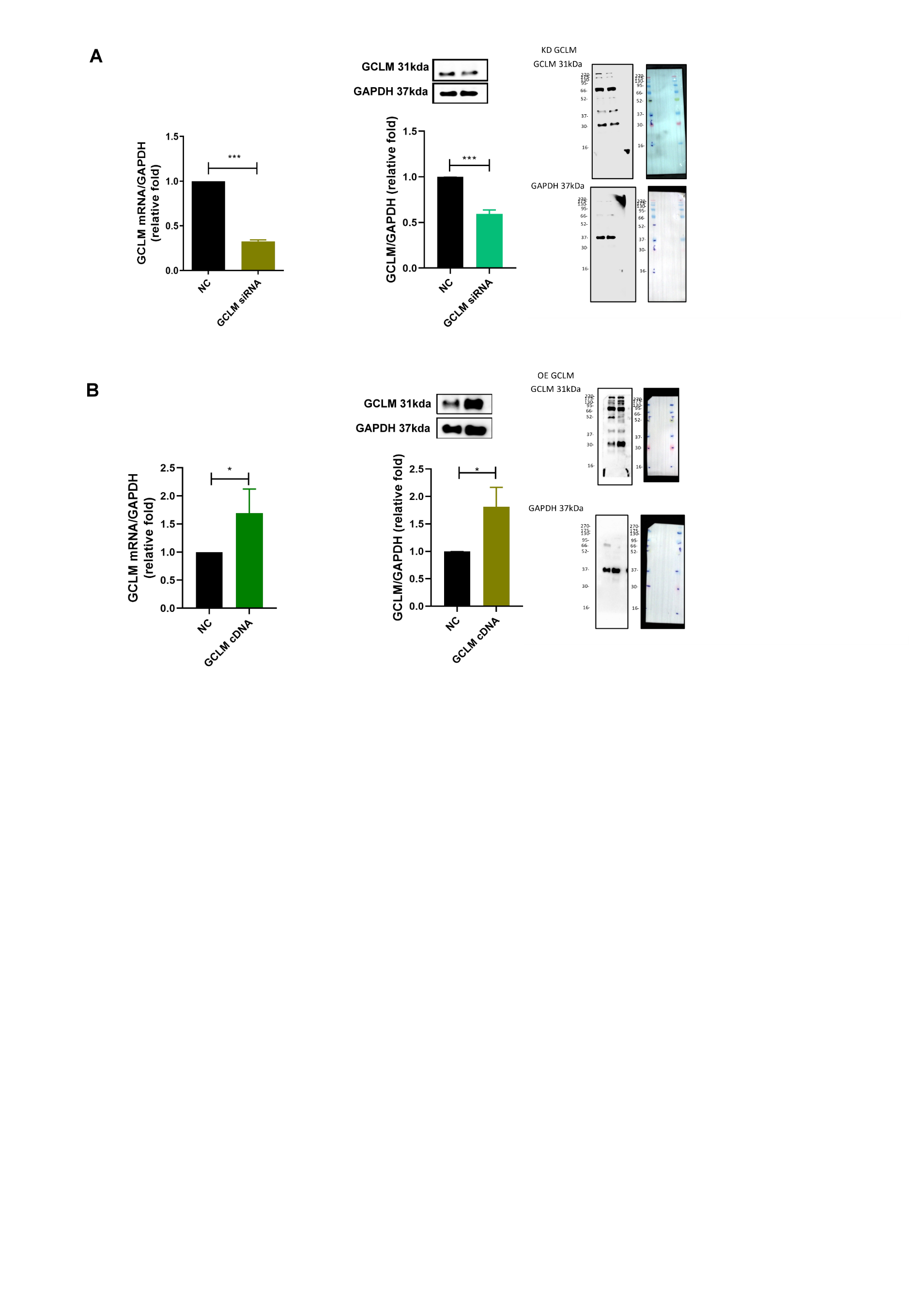

Supplement: supplemental materials_1140622.docx [file YRER_A_2528334_SM7208.docx]
